# Molecular Diagnostic Applications in Colorectal Cancer

**DOI:** 10.3390/microarrays3030168

**Published:** 2014-06-26

**Authors:** Laura Huth, Jörg Jäkel, Edgar Dahl

**Affiliations:** Molecular Oncology Group, Institute of Pathology, Medical Faculty of the RWTH Aachen University, Pauwelsstrasse 30, D-52074 Aachen, Germany; E-Mails: laura.huth@rwth-aachen.de (L.H.); jjaekel@ukaachen.de (J.J.)

**Keywords:** colorectal cancer, microarray, molecular diagnostics

## Abstract

Colorectal cancer, a clinically diverse disease, is a leading cause of cancer-related death worldwide. Application of novel molecular diagnostic tests, which are summarized in this article, may lead to an improved survival of colorectal cancer patients. Distinction of these applications is based on the different molecular principles found in colorectal cancer (CRC). Strategies for molecular analysis of single genes (as *KRAS* or *TP53*) as well as microarray based techniques are discussed. Moreover, in addition to the fecal occult blood testing (FOBT) and colonoscopy some novel assays offer approaches for early detection of colorectal cancer like the multitarget stool DNA test or the blood-based *Septin 9* DNA methylation test. Liquid biopsy analysis may also exhibit great diagnostic potential in CRC for monitoring developing resistance to treatment. These new diagnostic tools and the definition of molecular biomarkers in CRC will improve early detection and targeted therapy of colorectal cancer.

## 1. Introduction

**Figure 1:**
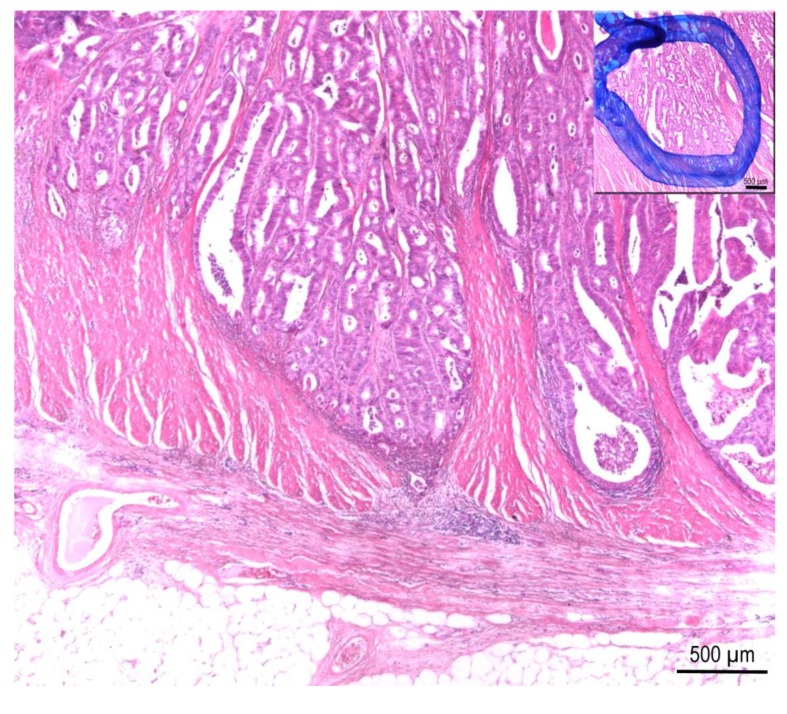
Colorectal cancer specimen, intermediate grade, showing invasion of *Tunica muscularis*. Insert: Labelled area defines tumor cells for manual microdissection. H&E stain, 20× magnification.

Globally more than 1.2 million new cases of colorectal cancer (CRC) are reported every year resulting in ~600,000 deaths [1]. Therefore, CRC is a major cause of cancer morbidity and mortality worldwide. The risk to get CRC rises with increasing age: 90% of new cases occur in people who are 50 years or older. On average, the individual risk for CRC is about 1 in 20, although this varies widely according to individual risk factors *i.e.*, intestinal polyps. The basis for all molecular analyzes of CRC is the exact definition of the affected tissue and subsequently the microdissection of an area enriched in tumor cells (Figure 1). The prognosis of survival is dependent on the stage of disease at diagnosis. CRC is divided into Union for International Cancer Control (UICC) stage I–IV cancers. Patients with UICC stage I colon cancer have an excellent 5-year survival rate of 90%, UICC stage II cancer patients present a survival rate between 70%–90%, whereas the 5-year survival rate decreases to 30%–90% in patients with UICC stage III tumors [2,3]. Adjuvant therapy is widely considered the standard of care in patients with UICC stage III CRC. However, the role of this therapy is controversial in stage II patients because the overall benefit is small [3].

This review article summarizes the current status and the perspectives of clinical applications of predominantly microarray-based assays in colorectal cancer. There are many promising applications which will support early detection and targeted therapies in colorectal cancer. They are summarized in Table 1. Subdivisions of these potential applications are based on various molecular states found in CRC that could serve as a basis for CRC clinical diagnostics. Application of these novel diagnostics tests may lead to an advancement of targeted therapies in personalized CRC oncology. Examples of developing commercially available assays are also discussed.

**Table 1:** Overview of molecular biology tests used in colorectal cancer (CRC) diagnostics.

Name/Method Target	Intended Use	Detected Property	Source Material	Molecular Method	Use/Availability
KRAS	therapeutic decision EGFR targeted therapy	KRAS mutations	FFPE or snap frozen tissue	Sequencing	clinical routine
KRAS	therapeutic decision EGFR targeted therapy	KRAS mutations	FFPE or snap frozen tissue	SnaPshot/strip assay, COLD-PCR, ARMS, PNA clamping; Digital PCR	studies
BRAF	chemotherapeutic susceptibility	BRAF mutations	FFPE or snap frozen tissue	sequencing, Real-time PCR	clinical routine
BRAF	chemotherapeutic susceptibility	BRAF mutations	FFPE or snap frozen tissue	Digital PCR, COLD-PCR	studies
MSI status PCR	chemotherapeutic susceptibility	MSI status	FFPE or snap frozen tissue	PCR	clinical routine
MSI status IHC	chemotherapeutic susceptibility	MSI status	FFPE tissue	IHC	clinical routine
MSI status 64 gene signature	chemotherapeutic susceptibility	MSI status	FFPE or snap frozen tissue	microarray	studies
MSI status miRNA	chemotherapeutic susceptibility	MSI status	FFPE or snap frozen tissue	oligonucleotide microarray	studies
TP53 mutation	screening	p53 mutation analysis	FFPE or snap frozen tissue	sequencing	clinical routine
TP53 mutation	screening	p53 mutation analysis	FFPE or snap frozen tissue	oligonucleotide microarray	studies
CIMP	probable screening/staging	methylation	FFPE or snap frozen tissue	methylation microarray	studies
Colo Print^®^	outcome and/or relapse prediction	18-gene expression signature	fresh tumor tissue	Agilent 44K oligonucleotide arrays	studies
CRC subtype gene expression profile	chemotherapeutic susceptibility, subtyping	gene signature, intended for IHC/qRT-PCR	tumor tissue	sequencing, IHC	studies
NGS	therapeutic decision EGFR targeted therapy; subtyping	driver mutations	tumor tissue	parallel sequencing	studies
multiplex-serum protein array	screening	serum markers	blood serum	protein array	studies
miRNA assay for blood/stool	screening	miRNA expression level	plasma, stool	micro array	studies
Multitarget stool DNA test	screening, increasing sensitivity for colonoscopy	KRAS mutation, NDRG4, BMP3 methylation, hemoglobin immunoassay	stool	mutation and methylation analysis, immunoassay	under approval for clinical use
Epi proColon early detection assay	screening, increasing sensitivity for colonoscopy	Septin 9 DNA methylation assay	blood plasma	Real-time PCR	available for clinical use

CIMP = CpG island methylator phenotype, FFPE = formalin fixed paraffin embedded, IHC = immunohistochemistry, miRNA = micro ribonucleic acid, MSI = microsatellite instability, PCR = polymerase chain reaction.

## 2. Activation of the Proto-Oncogenes *KRAS* and *BRAF* in Colorectal Cancer

Somatic mutations of the *KRAS* gene are found in approximately 40% of CRC [4]. KRAS is a member of the RAS superfamily, a family of monomeric small-G proteins, and functions as a transmitter of key extracellular signals (e.g., epidermal growth factors) into intracellular signal cascades [5]. Patients with CRC tumors expressing mutant *KRAS* will not benefit from a targeted therapy against EGFR [6]. Therefore mutation analysis of the *KRAS* gene is important in clinical practice supporting therapy decision. Although direct sequencing is the gold standard for the detection of *KRAS* mutations in clinical diagnostics, it remains laborious and is not very sensitive [7]. SNaPshot and reverse hybridization StripAssay were tested as alternatives to direct sequencing for *KRAS* mutation detection in daily routine. Detection limits of direct sequencing, SNaPshot and StripAssay were 20%, 10% and 1%, respectively [7]. Consequently, the authors concluded that SNaPshot and the StripAssay could both be alternatives to direct sequencing, especially in small laboratories without dedicated equipment. However, the risk of false positives is increased. Another study compared the performance and reagent costs of two new sensitive methods, a peptide nucleic acid (PNA) clamp PCR and amplification refractory mutation system PCR assay, for detection of KRAS mutations. The PNA clamping assay had a higher sensitivity as well as 20 times lower costs compared to ARMS [8]. Therefore, the authors concluded that due to high performance and low costs the PNA clamping assay could be a suitable method for detecting *KRAS* mutations.

BRAF is a serine-threonine protein kinase and a member of the RAF kinase gene family that acts as a downstream effector of the *KRAS* gene. Mutations of the *BRAF* gene, which were found at an early stage of CRC carcinogenesis, occur in 5%–15% of overall CRC [9]. Moreover, BRAF mutations appear to be a valid indicator of poor survival in patients with CRC [10]. Direct sequencing is the gold standard for the detection of *BRAF* mutations. Nevertheless, Benlloch and colleagues suggest a real-time PCR based test as a suitable alternative to direct sequencing. This test is highly sensitive and specific for detecting V600E mutations and has advantages in cost, time and labor [11]. Recently, the use of digital PCR to detect *BRAF* mutations was also mentioned [12]. This new technology is an attractive tool due to the formidable accuracy of results, time to results and cost per sample. In a further study, the use of COLD-PCR, a modified PCR protocol that allows preferential amplification of minority alleles from a mixture of wildtype and mutation-containing DNA, improves the detection limit of *KRAS* and *BRAF* mutations in CRC without requiring expensive and time-consuming procedures [13].

## 3. Detection of Patients with Microsatellite Instability Phenotype

CRC can be molecularly divided into two major subgroups, microsatellite-stable (MSS, ~85%) and microsatellite-instable (MSI, ~15%) CRCs. CRC patients presenting MSI have a better prognosis associated with a longer overall survival and a more benign disease course but their tumors are resistant to a wide range of chemotherapeutics [14,15]. Genes that correlate with MSI status were identified using full-genome expression data [16]. Subsequently, a MSI gene expression signature of 64 genes was developed and translated to a diagnostic microarray. This signature was able to identify patients with MSI status with high accuracy and additionally MSI-like patients who are not recognized by traditional methods like PCR or immunohistochemistry (IHC) [16]. The 64-gene signature owns some more advantages compared to PCR or IHC, *i.e.*, robust and reproducible measurements, the signature can be read out from the same tissue biopsy and does not require a comparison of regions from paired normal and tumor tissue [16]. The MS status of CRC could be also correctly predicted based on miRNA expression profiles. Spotted locked nucleic acid (LNA)-based oligonucleotide microarrays were used to profile the expression of 315 miRNAs [17]. Therefore, miRNAs may also be potentially used to classify colon cancers as either MSI or MSS.

## 4. Somatic TP53 (p53) Mutations in Colorectal Cancer

The p53 protein functions as a key transcriptional regulator in cell cycle regulation, apoptosis, gene transcription, DNA repair and angiogenesis [18,19,20]. Loss of wildtype *TP53* function facilitates the continued growth and the acquisition of invasive properties [21]. Mutations within the *TP53* gene are the most frequent genetic alterations in human cancer such as CRC. The GeneChip p53 assay is based on the recently developed oligonucleotide microarray technology. By screening primary colon cancer samples the assay was able to detect *TP53* mutations in 65% of tumors [22]. Direct sequencing confirmed the presence of these *TP53* mutations. By comparing the two methods, the GeneChip assay revealed several advantages, *i.e.*, higher throughput, higher sensitivity for point mutations and less expenditure of work [23,24]. In contrast the inability to detect deletions or insertions >1 bp and frameshift mutations demonstrates the limitation of the GeneChip assay. Nevertheless, GeneChip has an adequate sensitivity to detect *TP53* point mutations in primary colon cancers. Takahashi and colleagues concluded that the GeneChip p53 assay could be applicable to screening procedures in clinical samples but this potential application has to be confirmed in follow-up studies [22].

## 5. Enabling Diagnostic Technologies Based on Epigenetic Changes and Post-Translational Modifications in CRC

CRC develops in a multistep process that arises from genetic or epigenetic alterations. DNA methylation within a gene promoter and alterations in histone modifications appear to be primary mediators of epigenetic inheritance in cancer cells [25]. Cancers with high degrees of methylation (the CpG island methylator phenotype, CIMP) represent a clinically distinct group that is characterized by epigenetic instability [26]. In CRC CIMP is one of the underlying mechanisms in the development of tumors. Therefore, a methylome signature in CRC should be defined using a methylation microarray analysis (Illumina HumanMethylation27 array). This will lead to identification of a defined list for methylome specific genes that will be hopefully able to contribute to better clinical management of CRC patients in the future [27]. Changes in post-translational modifications (PTMs) can be also associated with cancer. Thereby, O-linked glycosylation represents one of the most important cancer-associated PTMs. Using novel glycopeptide microarrays a set of aberrant glycopeptides was identified. Using seromic profiling this array was able to detect CRC with a sensitivity of 79% and a specificity of 92% [28]. Therefore, the methodology to examine PTMs illustrates a fruitful and previously unaddressed source of sensitive biomarkers. Further expansion of this study should improve specificity and sensitivity before starting clinical trials.

## 6. Prediction of Disease Relapse in Stage II CRC Patients

Outcomes for patients with early-stage CRC are heterogeneous. Therefore, based on Agilent 44K oligonucleotide arrays an 18-gene expression signature was defined to predict the risk of disease relapse and development of distant metastasis of stage II patients [3,29]. This assay is called ColoPrint^®^ and became commercially available. Based on the mRNA expression level of the 18 gene signature this molecular diagnostic test identifies the patient’s risk of distant, local or regional relapse for stage II CRC patients. With a very high precision of 97.9% in reproducibility and analytical accuracy the information helps physicians to decide on appropriate treatment options. Several independent studies confirmed ColoPrint^®^ as a significant and strong factor for prediction of recurrence in comparison to all relevant clinical factors (age, gender, localization of tumor, grade, lymph node- and T-stage) [3,29]. In conclusion, ColoPrint^®^ represents an example of how microarray-based technology can be successfully used in clinical applications.

## 7. Defining CRC Subtypes Based on Gene Expression Profiles

The biological and clinical diversity of CRC makes it difficult to decipher which patients benefit and respond well to adjuvant therapy [30]. Therefore, it is important to get more insight into the heterogeneity of CRC to develop individualized treatment strategies. By analyzing gene expression profiles from 1290 CRC tumors six clinically relevant CRC subtypes were recently defined as follows: goblet-like, enterocyte, stem-like, inflammatory, cetuximab-sensitive transit-amplifying (CS-TA) and cetuximab-resistent transit-amplifying (CR-TA) [31]. These subtypes are phenotypically distinct in their disease-free survival (DFS) and diversify in degree of response to chemotherapy. A development of clinically applicable assays for subtype-specific signatures and of subtype-specific therapies could lead to an effective fight against this disease. At the same time Melo and colleagues defined three subtypes of CRC using an unsupervised classification strategy of 1100 gene expression profiles. Besides chromosomal-instable and microsatellite-instable cancers they observed a third subtype, which is largely microsatellite-stable. An up-regulation of matrix remodeling and epithelial-mesenchymal transition genes accompanied by poor prognosis and low therapy response was shown for this CRC subtype [32]. Classification of tumors into these different subtypes led to the identification of applicable biomarkers that might be developed into clinical qRT-PCR or immunohistochemical assays. Thereby, CRC tumors could be classified into one of these subtypes guiding to the assignment of subtype-specific therapeutic agents.

## 8. microRNAs in CRC

microRNAs (miRNAs) are a class of small non-coding single-stranded RNAs with important posttranscriptional regulatory functions. They have been found in various types of cancers and seem to play a role during the pathogenesis of CRC by binding to their target mRNAs which are protein-encoding [33]. Within the last years, several specific miRNAs were identified as predictive or prognostic biomarkers in CRC. For example, Dong and colleagues identified an involvement of miRNA-133a in regulating the EGFR pathway proposing an essential role in predicting the response to EGFR inhibitors [34]. Another recently published study showed that the expression of miRNA-146a and miRNA-147b can be used as a biomarker for colorectal tumor’s localization [35]. 

## 9. Detection of Circulating Tumor DNA in CRC

Apoptotic or necrotic dying tumor cells are thought to release short DNA fragments into the bloodstream that contain tumor-specific genomic alterations. This cell free circulating tumor DNA (ctDNA) can be easily collected non-invasively via blood samples from CRC patients [36]. Recently, it was demonstrated that ctDNA analysis is a powerful tool to monitor development of drug resistance in patients undergoing therapy with EGFR inhibitors [37]. The authors could show that >95% of patients that originally responded to anti-EGFR therapy but subsequently relapsed developed mutations in genes involved in the mitogen-activated protein kinase pathway. Thus, ctDNA mutation analysis may become an effective biomarker in the management of CRC. However, before it can enter into clinical routine, further studies are needed on the source of ctDNA and the robustness of its analysis.

## 10. Future Application of Next Generation Sequencing in CRC

Use of next generation sequencing (NGS) enables sequencing analysis of a whole individual tumor genome. This technology parallelizes the sequencing process, producing thousands or millions of sequences concurrently. Based on the lack of required analysis resources and understanding of novel mutations, whole genome sequencing will not become a routine diagnostic tool for cancer patients in daily practice in the next years (apart from clinical trials). However, simultaneous sequencing of gene panels by NGS analyzing all relevant driver mutations of CRC in parallel will be essential in clinically routine in the next years.

## 11. Early Detection of CRC

Unfortunately, the majority of CRC is not detected early. About 50% of the CRC patients are diagnosed at advanced tumor stages presenting poor prognosis [2]. Therefore, early screening for CRC has become one of the greatest public health challenges over the last fifty years. Development of genomic signatures that can be used for diagnosis and prognosis will be of interest, because many screening tests are invasive and/or expensive [38]. Currently established screening tests are fecal occult blood testing (FOBT) and colonoscopy. However, the sensitivity for detecting adenomas with FOBT is very low [39]. Moreover, the compliance for colonoscopy is quite low because it is time consuming, disturbing, painful and involving some risk [40]. A CRC screening test that accurately detects advanced adenomas with a high potential of malignant progression would be desirable [41]. Consequently, innovative screening tools are necessary for the detection of a pre-cancer condition and very early-stage malignancies in a healthy population allowing curative treatment interventions. A first excellent example may become the recently developed non-invasive multitarget stool DNA test (Cologuard™) for colorectal cancer screening that includes besides *KRAS* mutation analysis, detection of the methylation status of the *NDRG4* and *BMP3* genes. Indeed, this novel test was able to detect advanced precancerous lesions with much higher sensitivity than fecal immunochemical test (FIT) (42.4% *vs.* 23.8%, *p* < 0.001) [42]. The blood-based *Septin 9* DNA methylation assay (Epi proColon) represents another example which has shown that DNA methylation markers can be of great importance for early detection of CRC. Using highly sensitive real-time PCR aberrantly methylated tumor-derived DNA of the *Septin 9* gene can be detected in blood plasma [43]. One recent approach for serum based detection of CRC uses biochip array technology [2] applying competitive and sandwich assay techniques using antibodies as capture molecules [44]. For this purpose, a multiplex serum protein biochip array was developed for the determination of nine serum markers. Significant differences between cases and controls were observed in serum levels of these markers [2]. It was the first study reporting the development of a multiplex protein array for clinical application to CRC screening. Advantage of this array is that the required sample volume is very low and the throughput is high: 1200 samples can be simultaneously analyzed for the nine serum markers in one hour using a fully automated biochip immunoassay analyzer. [44]. A combination of FOBT and the biochip array may improve the performance of CRC screening because neither compliance nor diagnostic performance of both methods alone seems to be satisfying. Another approach for developing blood serum based diagnostic screening test for early detection of CRC used the expression of miRNAs. Using a global microarray, characteristic changes in the expression level of miRNA molecules in plasma were discovered between CRC patients and healthy controls [45]. Additionally, miRNA expression alterations were also observed within stool samples by use of microarray expression studies [41]. Both plasma and stool samples seem well-suited for screening processes [41]. Analysis of miRNA molecules has opened new opportunities for a quantitative and non-invasive diagnostic approach for CRC screening in the future. Research groups are standardizing test conditions in a prospective validation study. The goal is to develop a chip-based diagnostic test that facilitates molecular screening for CRC [41]. Moreover, the GenomeLab Genetic Analysis System presents a novel technology platform for custom design of multiplexed gene expression analysis (GeXP assay). Only nanograms of total RNA are necessary to allow exact quantification of multiple targets simultaneously in a single reaction [46]. 

## 12. Conclusions

In the last decade, the median of CRC patient’s survival has increased significantly (~20%) with the introduction of new routine diagnostics and personalized therapies. Notably, the definition of molecular markers has led to important advances in the personalized treatment of colorectal cancer. It is obvious that determination of molecular predictive factors analyzed in routine diagnostics before selection of chemotherapy is the exemplar of individualized treatment of colorectal cancer. Moreover, new analytical methods such as next generation sequencing will help to enlarge the knowledge of potential biomarkers in the future. This method shows that Moore’s law is obsolete and it represents a quantum leap helping an aging society to fight against the diseases of age. There is a long way to go to defeat colorectal cancer but the new techniques presented in this article help us on this journey.
